# Transareolar uniportal thoracoscopic extended thymectomy for patients with myasthenia gravis

**DOI:** 10.3389/fsurg.2022.914677

**Published:** 2022-10-11

**Authors:** Jianbo Lin, Nanlong Lin, Xu Li, Fancai Lai

**Affiliations:** ^1^ Department of Thoracic Surgery, Palmar Hyperhidrosis Research Institute, The First Affiliated Hospital of Fujian Medical University, Fuzhou, China; ^2^ Fujian Key Laboratory of Precision Medicine for Cancer, The First Affiliated Hospital, Fujian Medical University, Fuzhou, China

**Keywords:** transareolar uniportal thoracoscopic extended thymectomy, myasthenia gravis, areola, feasibility, safety

## Abstract

**Background:**

Transareolar uniportal thoracoscopic extended thymectomy (TUTET) has not been previously reported. We attempted to assess the feasibility and safety of TUTET for male myasthenia gravis (MG) patients.

**Patients and methods:**

From February 2013 to February 2020, 46 men with MG underwent TUTET. All patients were followed up for 12–84 months postoperatively by clinic visits or telephone/e-mail interviews.

**Results:**

All surgeries were completed successfully, with an average operation time of 72.6 min. The mean length of transareolar uniportal incision was 3.0 ± 0.4 cm, and the mean postoperative cosmetic score was 3.1 ± 0.5 at discharge. Three months postoperatively, no patients had an apparent surgical scar on the chest wall or complained of postoperative pain. Substantial amelioration of the disease was achieved in a short period, and several benefits were clear. At the 1-year follow-up, all patients showed a good cosmetic effect and high satisfaction.

**Conclusions:**

TUTET is an effective and safe way for men with MG. The uniportal incision is hidden in the areola with sound cosmetic effects. We believe that TUTET is an acceptable procedure for extended thymectomy.

## Introduction

Extended thymectomy is a therapeutic avenue for myasthenia gravis (MG) with thymic hyperplasia or thymoma, yielding favorable outcomes (1–5). Trans-sternal extended thymectomy, categorized as T3b by the Myasthenia Gravis Foundation of America, was considered the gold standard approach for several decades (6). However, video-assisted thoracic surgery (VATS) has since been prevalent (7). Bilateral video-assisted thoracoscopic extended thymectomy (VATET) has been performed in many institutions with assorted superiorities over trans-sternal thymectomy, such as less tissue injury, a shorter length of hospital stay, and better cosmetic results (8). Unilateral VATET has long-term outcomes comparable with those of bilateral VATET, but it reduces the number of incisions and operating time (6).

Unilateral VATET is often performed by conventional three-port VATS (cVATS). However, 3 visible scars left on the chest wall by cVATS cause a permanent cosmetic defect. Performing multiple incisions through different intercostal spaces occasionally stimulates intercostal nerve injury, which leads to paresthesia or numbness and is accompanied by wound-related pain postoperatively (9). Single-port VATS as an approach with minimum invasiveness is used to reduce postoperative pain, the length of hospitalization and residual paresthesia in comparison with multiport VATET (10), but the thoracic incision is still evident and may last a lifetime.

In February 2013, we developed transareolar uniportal thoracoscopic extended thymectomy (TUTET) for patients with MG at our hospital. We herein reported our initial experience with TUTET for MG.

## Patients and methods

### Patient selection

From February 2013 to February 2020, 46 men with MG underwent TUTET at our institution. MG was diagnosed upon a history of fatigable weakness, a positive response to the anticholinesterase test, and aberrant test outcomes of repetitive nerve stimulation or single-fiber electromyography. The Myasthenia Gravis Foundation of America clinical classification was used for assessment of disease severity (11).

Patient characteristics are listed in Table 1. This work received approval from the Institutional Review Board and Ethics Committee of the First Affiliated Hospital of Fujian Medical University. All patients underwent a preoperative routine blood examination, acetylcholine receptor antibody assay, electrocardiography, echocardiography, pulmonary function measurement, and chest computed tomography (CT) scan. The quantitative MG score (QMG score) (11) was calculated for objective evaluation of MG. All patients provided written informed consent before surgery after receiving a detailed explanation of procedure and aims of the study.

**Table 1:** Patient characteristics.

Characteristics	Value
No. of patients	46
Mean age/range (years)	38.6 (19–60)
Positive family history (yes/no)	1/46
Preoperative dosage of bromopirix, *n* (%)	
60 mg q8h	37/80.4
60 mg q6h	8/17.4
90 mg q8h	1/2.17
Preoperative dosage of prednisone, *n* (%)	
>30 mg qd	2/4.35
≤30 mg qd	44/95.7
MGFA clinical classification, *n* (%)	
I	14/30.4
IIa	18/39.1
IIb	7/15.2
IIIa	5/10.9
IIIb	1/2.17
Iva	1/2.17
Pathologic characteristics	
thymic hyperplasia	18/39.1
thymic cyst	9/19.6
thymoma	
type A	15/32.6
type AB	3/6.52
type B1	1/2.17

Inclusion criteria were an age of >18 to <60 years; male sex; MG with thymic hyperplasia or thymoma (tumor diameter of <2 cm, without invasion of the mediastinum or metastasis); and, for smokers, no smoking for ≥2 weeks before operation. The exclusion criteria were an age of <18 or >60 years; female sex; obesity (body mass index >30 kg/m^2^); history of thoracic surgery or severe chest wall deformity; upper airway and maxillofacial injury or deformity; and lung, heart, or pleural disease.

### Surgical procedure

**Figure 1:**
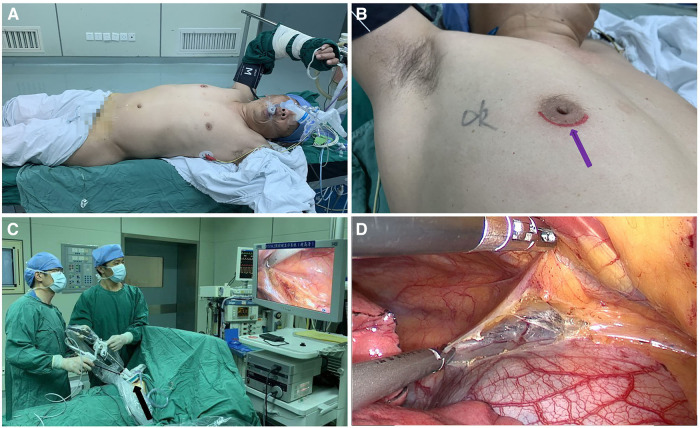
(**A**) the patient was placed in the left lateral decubitus position at 45° during surgery. (**B**) Transareolar uniportal incision was marked before the operation. The purple arrow indicates the lower edge of the areola. (**C**) Procedure of transareolar uniportal thoracoscopic extended thymectomy for patients with myasthenia gravis. (**D**) Transareolar uniportal endoscopic view of the right thoracic cavity.

**Figure 2:**
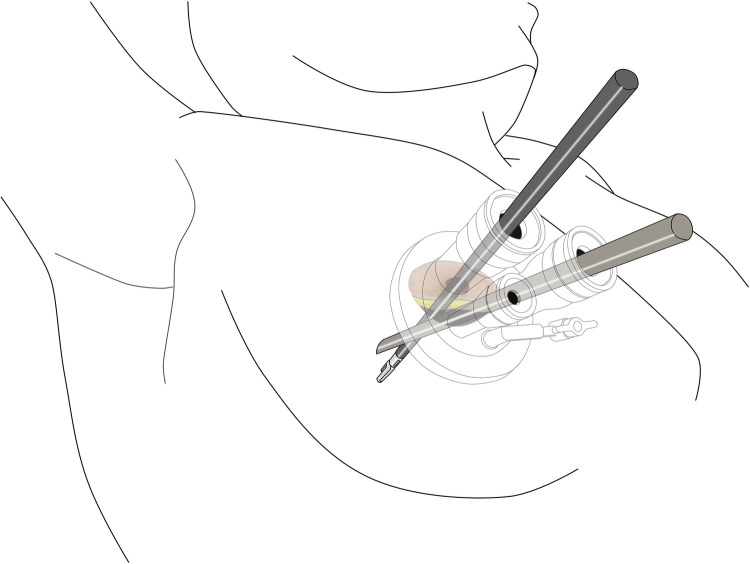
Procedure of TUTET for patients with MG.

**Figure 3:**
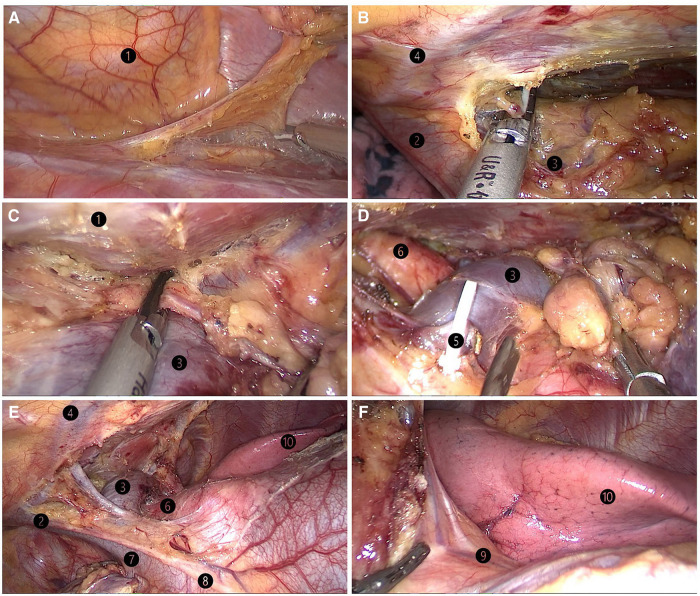
(**A**) lifting of the lower pole of the thymus with the grasping forceps. (**B**) Dissection of the right upper thymic lobe. (**C**) Dissection of the left upper thymic lobe. (**D**) Resection of the thymic vein. (**E**) Skeletonization of both innominate veins, the superior vena cava, and the aorta. (**F**) Left lung and left phrenic nerve. ❶ sternum; ❷ right brachiocephalic vein; ❸ left brachiocephalic vein; ❹ internal thoracic artery and vein;❺ thymic vein; ❻ aorta ascendens; ❼ superior vena cava; ❽ right phrenic nerve;❾ left phrenic nerve; ❿ left lung.

**Figure 4:**
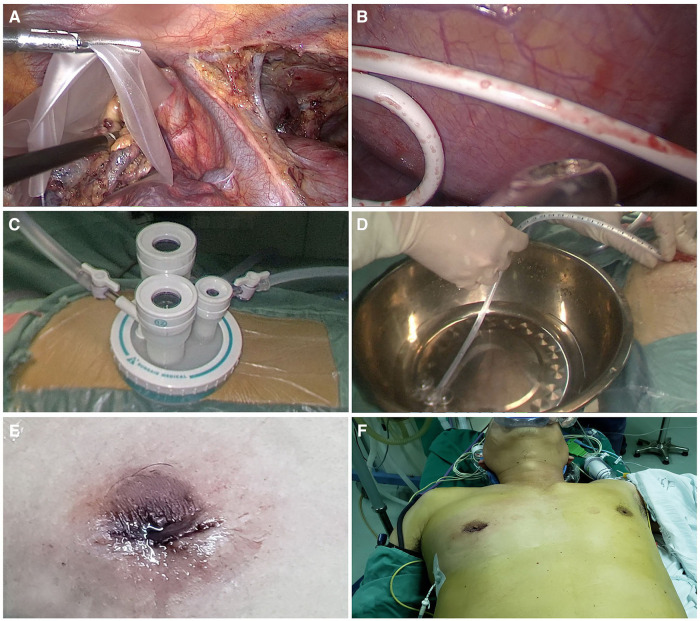
(**A**) placement of specimens in a plastic bag. (**B**) Placement of a 2-mm subclavian vein tube through the sixth intercostal space at the midaxillary line. (**C**) Uniportal wound protector. (**D**) Ventilation and exertion of continuous positive pressure for a few seconds. (**E,F**) Postoperative view of transareolar uniportal incision.

All patients underwent surgery in the left lateral decubitus position at 45° the operating table, with the right arm abducted in a holder above the head (Figure 1A). Transareolar uniportal incision was marked before the operation, and then the pocedure of transareolar uniportal thoracoscopic extended thymectomy was performed (Figures 1B-D). Following general anesthesia, the patient was ventilated through a single-lumen endotracheal tube. A 30-mm single port was made through the fourth intercostal space at the lower edge of the areola (Figure 2). A 10-mm 30° thoracoscope (Karl Storz GmbH / Co. KG, Tuttlingen, Germany) was introduced for observation of thoracic cavity. A wound protector (Endo Keeper; Hangzhou Kangji Medical Instrument Co., Ltd., Hangzhou, China) was fixed on the incision window. After the areola was covered with a multichannel thin-film puncture kit, 8 cm H_2_O of positive-pressure carbon dioxide was insufflated to create artificial pneumothorax, so as to expose the phrenic nerve. To avoid phrenic nerve injury, the thymus and prepericardial adipose tissue were generally removed at a distance of 0.5–1.0 cm from the phrenic nerve, and energy instruments such as ultrasonic knife rather than electrocoagulation hook were used as far as possible. The whole thymus with or without the thymoma, the bilateral mediastinal pleura, and all associated fat tissue between the two phrenic nerves were excised *en bloc*. Grasping forceps was used to lift the lower poles of the thymus (Figure 3A), exposing the superior vena cava, ascending aorta, and innominate veins. Second, thymus was pulled down and the two upper thymic lobes were dissected meticulously (Figures 3B,C). Third, by tracing the superior vena cava and the innominate veins, all of the thymic veins were easily identified and resected (Figure 3D). Harmonic scalpel was utilized to simultaneously dissect all of the fat pads near the phrenic nerves. The prepericardial fat and the fat pads near the cardiophrenic angle, aortopulmonary window, and lower poles of the thyroid gland were dissected and removed carefully. Both innominate veins, the superior vena cava, and the aorta were skeletonized, and then the left lung and left phrenic nerve were exposed (Figures 3E, F). All specimens were placed in a plastic bag and removed *via* the single port (Video1, Figure 4A). A 2-mm subclavian vein tube was placed through the sixth intercostal space at the midaxillary line (Figure 4B). Before the end of the operation, we would completely stop bleeding on the surgical wound. If there was no special risk of bleeding, we usually only drained one side of the chest. All procedures were completed successfully, and the lung was re-inﬂated under visual control. The incision was closed while the anesthesiologist manually ventilated the patient, exerting continuous positive pressure for a second (Figures 4C,D). Following the suture of muscle and subcutaneous tissue, the wound was dressed with a skin adhesive (Dermabond; Ethicon, Inc., Cincinnati, OH, USA) (Figures 4E,F). Postoperative chest radiography was generally taken to see whether there is abnormal elevation of diaphragm muscle to determine whether there is phrenic nerve injury.

The patient had always taken pyridostigmine bromide before surgery (60 mg), 3–4 times/day with the maximum dose in the whole day no more than 480 mg. For glucocorticoids such as prednisone, it is best to adjust glucocorticoids to the lowest effective dose (daily dosage ≤30 mg) or stop taking them completely before operation. Adjust the dosage according to the improvement of symptoms after operation (It is generally recommended to use drugs for follow-up in neurology outpatient department).

### Data collection and follow-up

We recorded operation time, incision length, intraoperative bleeding, complications, pain score and hospital stay. All patients were followed up by hospital visits for 12 to 84 months with the mean follow-up duration of 26.8 ± 6.2 months. All out of them completed a detailed questionnaire (Table 2). Postoperative dyspnea, satisfaction scores, QMG score, cosmetic scores, residual pain, and incidence of tumor recurrence or metastasis were collected. Tumor recurrence was assessed by chest CT. Patients were asked to routinely undergo a chest CT scan every 3 months and a systematic general inspection every 6 months during follow-up. Surgical outcomes were graded in line with the criteria used by MGFA Postintervention Status (11): 1, Complete Stable Remission (CSR); 2, Pharmacologic Remission (PR); 3, Minimal Manifestations (MM); 4, Improved (I); 5, Unchanged (U); 6, Worse (W); 7, Exacerbation (E); and 8, Died of MG (D of MG).

**Table 2:** Follow-up questionnaire.

Nature of question
Response
1. Cosmetic results
Verbal response scale (VRS): 1 dissatisfied/2 accepted/3 satisfied/4 perfect
2. Postoperative pain
Visual analog scale (VAS) pain score: from 0 “no pain” to 10 “worst pain imaginable”
3. Postoperative QMG score
Scored items:ptosis, diplopia, facial weakness, dysphagia, dysarthria, neck muscle strength, bilateral arm muscle strength, bilateral hand grips, bilateral leg muscle strength, vital capacity. Each item was graded on a scale of 0–3 (0 normal, 1 mild, 2 moderate, and 3 severe)
4. Tumor recurrence or metastasis
No/Yes
Location: left lung/right lung/liver/epinephros/brain/bone/other
5. Surgical outcomes
Criteria used by MGFA Postintervention Status and associates: 1, Complete Stable Remission (CSR); 2, Pharmacologic Remission (PR); 3, Minimal Manifestations (MM); 4, Improved (I); 5, Unchanged (U); 6, Worse (W); 7, Exacerbation (E); and 8, Died of MG (D of MG).
6. Satisfaction
VAS: very satisfied (9–10)/satisfied (6–8)/dissatisfied (3–5)/very dissatisfied (0–2)

## Results

All procedures were completed successfully with an average operation time of 72.6 min (Table 3). No surgical mortality or intraoperative conversion to an open procedure occurred. The mean drainage time was 1.6 days, with a mean drainage amount of 63.4 ± 28.2 ml/day. The mean hospitalization time was 3.6 ± 1.6 days. The mean transareolar uniportal incision length was 3.0 ± 0.4 cm, and the mean postoperative cosmetic score was 3.2 ± 0.7 at discharge. Postoperative pathologic diagnoses were thymic hyperplasia (*n* = 18), thymic cyst (*n* = 9), type A thymoma (*n* = 15), type AB thymoma (*n* = 3), and type B1 thymoma (*n* = 1). One patient developed myasthenic crisis on postoperative day 3. After tracheal intubation with ventilator-assisted breathing and drying therapy, he recovered quickly and was discharged 2 weeks later. One patient (2.17%) developed postoperative pneumonia (Table 4). This patient a long-term heavy smoker, had stopped smoking just 2 weeks before surgery. He was treated with intravenous antibiotics and no intervention was required subsequently. None of the patients had wound infection. Postoperative clinical improvement was evaluated using the QMG score in all patients who were followed up for >12 months.

**Table 3:** Perioperative outcomes.

Characteristics	
Mean total operative time (min)	72.6 ± 17.2
Mean intraoperative bleeding (ml)	63.4 ± 28.2
Mean length of transareolar uniportal incision	3.0 ± 0.4
Mean drainage (ml/day)	63.4 ± 28.2
Mean drainage time (days)	1.6 ± 1.2
Mean postoperative hospital stay (days)	3.6 ± 1.6

**Table 4:** Postoperative complications.

Characteristics	*n* (%)
Myasthenic crisis	1 (2.17)
Wound infection	0
Arrhythmia	1 (2.17)
Pleural effusion	2 (4.35)
Permanent phrenic nerve injury	0
Hypoxia	0
Pneumonia	1 (2.17)
Atelectasis	0
Cerebrovascular accident	0

**Figure 5:**
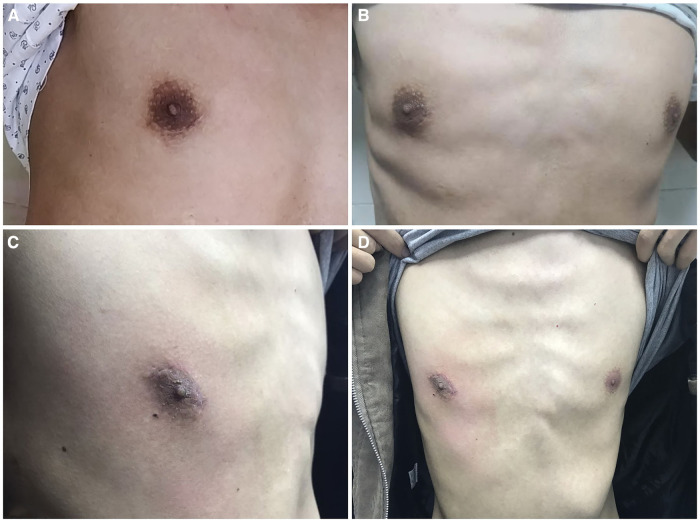
(**A,B**) the surgical scar was concealed in the areola 6 months after surgery. (**C,D**) Photograph of the same patient 8 months after surgery showing the absence of any obvious surgical scar on the chest wall.

Follow-up completion rate was 100%. Outcomes of postoperative follow-up are shown in Table 5. In patients who underwent TUTET, the mean aggregate QMG score decreased remarkably from 5.6 ± 3.8 preoperatively to 1.6 ± 1.2 at 6 months postoperatively. Complete remission (score of 1) was observed in 2.17% (1 of 46) of patients and clinical improvement (scores of 2 and 3) in 8.70% (4 of 46). No tumor recurrence or metastasis was observed. Five patients (10.9%) reported postoperative pain at discharge. Based on visual analog scale (VAS) pain score, mildly painful (mean VAS score of 2.09 ± 0.42) was considered in four patients (80%) and moderately painful (VAS score of 4) in one patient (20%). None of the patients required analgesia or complained of postoperative pain 3 months postoperatively. During follow-up, TUTET produced good surgical outcomes, excellent cosmetic results (Table 5 and Figures 5A–D), and a high degree of satisfaction (Table 5).

**Table 5:** Outcomes of postoperative follow-up.

Demographics	Discharge	6 months	12 months
Mean cosmetic score, VRS	3.1 ± 0.5	3.7 ± 0.4	3.8 ± 0.2
Degree of postoperative pain, *n* (%)			
Mild	4 (8.70)	0	0
Moderate	1 (2.17)	0	0
Severe	0	0	0
Mean postoperative QMG score	5.4 ± 3.6	1.6 ± 1.2	1.2 ± 0.6
Tumor recurrence or metastasis	0	0	0
Surgical outcomes, *n* (%)			
1 Complete Stable Remission (CSR)	0	1 (2.17)	2 (4.35)
2 Pharmacologic Remission (PR)	0	2 (4.35)	5 (10.9)
3 Minimal Manifestations (MM)	0	4 (8.70)	9 (19.6)
4 Improved (I)	0	39 (84.8)	30 (65.2)
5 Unchanged (U)	0	0	0
6 Worse (W)	0	0	0
7 Exacerbation (E)	0	0	0
8 Died of MG (D of MG)	0	0	0
Degree of satisfaction, *n* (%)			
Very satisfied	40 (87.0)	45 (97.8)	46 (100)
Satisfied	6 (13.0)	1 (2.17)	0
Dissatisfied	0	0	0
Very dissatisfied	0	0	0

## Discussion

MG is featured by production of antibodies to acetylcholine receptors at the neuromuscular junction (12), being responsible for most acquired neuromuscular junction disorders (13). Patients with MG experience debilitating fluctuating weakness of ocular, limb, swallowing, or respiratory muscles, and symptoms are exacerbated by overwork or stress. Research has displayed that the thymus plays a key role in the MG autoimmune pathogenesis, and thymectomy can improve symptoms and achieve complete remission of MG (14, 15). After the first report of thymectomy in 1941 (16), alternative avenues such as full median sternotomy, transcervical or partial sternotomy, VATET (17–20), infrasternal mediastinoscopic thymectomy (21), trans-subxiphoid VATS (22, 23), and robotic thymectomy (24) have been developed. VATET has become immensely popular on account of better cosmetic results, less tissue damage, shorter hospital stay, and lower incidence of myasthenic crisis versus traditional extended thymectomy (25–27). Unilateral VATET is the most frequently performed procedure and is recognized as the surgery of choice for thymectomy (28–31).

Although traditional VATET is effective for MG patients, this technique has some shortcomings. First, this procedure must be performed *via* three incisions. Making multiple incisions through different intercostal spaces induces more chest wall muscle damage and increases the chance of intercostal nerve injury, leading to postoperative wound-related pain, paresthesia or numbness (9). Second, cVATS inevitably leave multiple apparent scars on the chest wall, which is a permanent cosmetic defect. As MG is popular in young and middle-aged people, the cosmetic outcome is vital after thoracic surgery. Infrasternal mediastinoscopic thymectomy is favorable over the trans-sternal and transcervical approaches in the context of cosmetic benefits, but it has disadvantages such as a longer operation time and a requirement for special equipment to lift the sternum (21). Trans-subxiphoid VATET has the advantages of avoidance of intercostal nerve compression or injury and good exposure of the anterior mediastinum. However, because of the narrow space behind the sternum, this approach has disadvantages of operational difficulties and the requirement for a certain learning curve to achieve a stable operation platform (23). Moreover, if intraoperative hemorrhage occurs and emergency conversion to thoracotomy is needed, median sternotomy is more traumatic than the lateral thoracic approach through the ribs. Robotic thymectomy is not widely used because of the need for expensive machines and consumables. Additionally, the system takes a long time to assemble, lacks tactile feedback, and requires multiple incisions (32).

To achieve superior cosmetic outcomes of VATET, we developed TUTET for MG in which incision scar is hidden in the areola. If MG is complicated with thymoma and the main body of tumor is inclined to the right, the right thoracic approach is preferred. If the main body of tumor is inclined to the left, the left thoracic approach is preferred. We were motivated by a previously reported success of transareolar VATS sympathectomy for palmar hyperhidrosis using a single incision in 2011, which achieved both satisfying efficacy and perfect cosmetic outcomes (33).

We successfully performed TUTET for all 46 men with MG in the present study. This procedure is not recommended for women or obese men due to their progressed mammary glands or fatty chest wall (9). The procedure led to a high satisfaction rate with minimal postoperative pain, a short hospital stay, good surgical and cosmetic outcomes, no major complications, and no recurrence/metastasis within a minimal follow-up of 1 year. TUTET seems to have several potential advantages over traditional MG treatment techniques.

First, incision is hidden in areola, with inapparent scar on the chest wall after operation; thus, it is especially suitable for patients with darkly pigmented areolas (Figures 5A,B) (9). Second, the incision is in the fourth intercostal space at the front of the chest, where the muscle layer is thinner and intercostal space is broader than in the lateral chest; this facilitates operating flexibility (34). Third, one study showed that >50% of patients who underwent cVATS experienced postoperative paresthesia related to the incision (35). The transareolar approach has an enlarged operating space, which minimizes intercostal nerve injury risk and reduces postoperative paresthesia. Moreover, it reduces the number of incisions, which avoids damaging the thick back chest wall muscles and thus reduces surgical trauma and related postoperative pain. Fourth, double-lumen endotracheal intubation is essential in traditional VATET. However, this intubation technique has drawbacks like prolonged intubation time, the need for anesthesia expertise, airway trauma risk, and costs exceeding those of single-lumen tube intubation (36). The use of artificial pneumothorax with positive-pressure carbon dioxide not only enables retrosternal space enlargement and facilitates thymus dissection, but it also allows operation under single-lumen endotracheal intubation. Fifth, carbon dioxide enters the thoracic cavity under artificial pneumothorax, which can promote inflation in mediastinum and avail the exposure of the operation field. Carbon dioxide insufflation can also remove air, improve the safety of electric burning and reduce the risk of air embolism. Simple thoracoscopic extended thymectomy in the past has limitations for it is difficult to remove lateral mediastinal fat, and it is more difficult to operate for large and obese patients. Artificial pneumothorax makes the lungs collapse sufficiently, which provides more favorable conditions for surgical operation and improves the safety of operation. Although its application may cause some complications, such as affecting the patient's circulation and lung ventilation function during operation. Rapid insufflation may induce an increase in the concentration of carbonic acid in the blood, resulting in hypercapnia and subcutaneous emphysema after operation. However, at present, most surgeons agree that carbon dioxide artificial pneumothorax will bring more benefits. Sixth, uniportal VATS decreases the severe postoperative pain; hence, the pain caused by chest drainage is more prominent (37). Managing pain in patients with MG can be challenging because of the complexity of the disease and its particular susceptibility to adverse effects of drugs (38). The traditional large chest tube not only induces chest pain, obstructs breathing, and hinders early out-of-bed activities after the operation but also affects wound healing and aesthetics, which is becoming patients' main complaint after thoracic surgery. Herein, we utilized a 2-mm subclavian vein tube instead of the traditional large chest tube; this minimized invasiveness and pain, could be easily removed with a good cosmetic outcome. Finally, we believe that the use of a single 3-cm transareolar port avoids enlarging the incision during the process of specimen extraction in most cases (which is especially convenient for patients with thymic tumors) and shortens the operation time.

This study has two main deficiencies. First, it was completed in a single institution with small proportion of patients and was retrospective. Second, all patients were relatively young men with no cardiopulmonary comorbidities, which may have contributed to the good results of this study. Thus, prospective multicenter randomized controlled trials are warranted to confirm the benefits of TUTET for men with MG.

## Conclusion

This study revealed that TUTET is an effective and safe way for men with MG as driven by its sound cosmetic and clinical outcomes. TUTET may be a promising therapeutic approach for MG.

## Data Availability

The original contributions presented in the study are included in the article/Supplementary Material, further inquiries can be directed to the corresponding author/s.

## References

[1] Chen Z Luo H Peng Y Cai L Zhang J Su C Comparative clinical features and immune responses after extended thymectomy for myasthenia gravis in patients with atrophic versus hyperplastic thymus. Ann Thorac Surg. (2011) 91:212–8. 10.1016/j.athoracsur.2010.08.041 21172515

[2] Budde JM Morris CD Gal AA Mansour KA Miller JI Jr. Predictors of outcome in thymectomy for myasthenia gravis. Ann Thorac Surg. (2001) 72:197–202. 10.1016/S0003-4975(01)02678-9 11465178

[3] Ozdemir N Kara M Dikmen E Nadir A Akal M Yücemen N Predictors of clinical outcome following extended thymectomy in myasthenia gravis. Eur J Cardiothorac Surg. (2003) 23:233–7. 10.1016/S1010-7940(02)00744-3 12559348

[4] Masaoka A Yamakawa Y Niwa H Fukai I Kondo S Kobayashi M Extended thymectomy for myasthenia gravis patients: a 20-year review. Ann Thorac Surg. (1996) 62:853–9. 10.1016/S0003-4975(96)00376-1 8784019

[5] Wolfe GI Kaminski HJ Aban IB Minisman G Kuo HC Marx A Randomized trial of thymectomy in myasthenia gravis. N Engl J Med. (2016) 375:511–22. 10.1056/NEJMoa1602489 27509100 PMC5189669

[6] Liu Z Yang J Lin L Huang J Jiang G. Unilateral video-assisted thoracoscopic extended thymectomy offers long-term outcomes equivalent to that of the bilateral approach in the treatment of non-thymomatous myasthenia gravis. Interact Cardiovasc Thorac Surg. (2015) 21:610–5. 10.1093/icvts/ivv176 26254034

[7] Lee CY Kim DJ Lee JG Park IK Bae MK Chung KY. Bilateral video-assisted thoracoscopic thymectomy has a surgical extent similar to that of transsternal extended thymectomy with more favorable early surgical outcomes for myasthenia gravis patients. Surg Endosc. (2011) 25:849–54. 10.1007/s00464-010-1280-y 20721579

[8] Lin MW Chang YL Huang PM Lee YC. Thymectomy for non-thymomatous myasthenia gravis: a comparison of surgical methods and analysis of prognostic factors. Eur J Cardiothorac Surg. (2010) 37:7–12. 10.1016/j.ejcts.2009.05.027 19615918

[9] Lin JB Chen JF Lai FC Li X Qiu ML. Transareolar pulmonary bullectomy for primary spontaneous pneumothorax. J Thorac Cardiovasc Surg. (2016) 152:999–1005. 10.1016/j.jtcvs.2016.06.023 27496616

[10] Wu CF Gonzalez-Rivas D Wen CT Liu YH Wu YC Chao YK Single-port video-assisted thoracoscopic mediastinal tumour resection. Interact Cardiovasc Thorac Surg. (2015) 21:644–9. 10.1093/icvts/ivv224 26273069

[11] Jaretzki A 3rd Barohn RJ Ernstoff RM Kaminski HJ Keesey JC Penn AS Myasthenia gravis: recommendations for clinical research standards. Task force of the medical scientific advisory board of the myasthenia gravis foundation of America. Ann Thorac Surg. (2000) 70:327–34. 10.1016/S0003-4975(00)01595-2 10921745

[12] Kim AG Upah SA Brandsema JF Yum SW Blinman TA. Thoracoscopic thymectomy for juvenile myasthenia gravis. Pediatr Surg Int. (2019) 35:603–10. 10.1007/s00383-019-04441-0 30729982 PMC6456483

[13] Fan PM Chen GP Jiang CN Lv PF Li JT Chen ZL Modified unilateral video-assisted thoracoscopic extended thymectomy for myasthenia gravis using 5-mm incisions: a case report. Medicine (Baltimore). (2018) 97:e11237. 10.1097/MD.0000000000011237 30075494 PMC6081170

[14] Jaretzki A 3rd. Thymectomy for myasthenia gravis: analysis of controversies–patient management. Neurologist. (2003) 9:77–92. 10.1097/01.nrl.0000051446.03160.2e 12808370

[15] Gronseth GS Barohn RJ. Practice parameter: thymectomy for autoimmune myasthenia gravis (an evidence-based review): report of the Quality Standards Subcommittee of the American Academy of Neurology. Neurology. (2000) 55:7–15. 10.1212/WNL.55.1.7 10891896

[16] Blalock A Harvey AM Ford FR Lilienthal JL. The treatment of myasthenia gravis by removal of the thymus gland: preliminary report. JAMA. (1941) 117:1529–33. 10.1001/jama.1941.02820440037009

[17] Kirschner PA Osserman KE Kark AE. Studies in myasthenia gravis. Transcervical total thymectomy. JAMA. (1969) 209:906–10. 10.1001/jama.1969.03160190028006 5819465

[18] Kark AE Kirschner PA. Total thymectomy by the transcervical approach. Br J Surg. (1971) 58:321–6. 10.1002/bjs.1800580502 5574714

[19] Miller JI Mansour KA Hatcher CR Jr. Median sternotomy T incision for thymectomy in myasthenia gravis. Ann Thorac Surg. (1982) 34:473–4. 10.1016/S0003-4975(10)61416-6 7138118

[20] Scelsi R Ferrò MT Scelsi L Novellino L Mantegazza R Cornelio F Detection and morphology of thymic remnants after video-assisted thoracoscopic extended thymectomy (VATET) in patients with myasthenia gravis. Int Surg. (1996) 81:14–7. 8803698

[21] Uchiyama A Shimizu S Murai H Kuroki S Okido M Tanaka M. Infrasternal mediastinoscopic thymectomy in myasthenia gravis: surgical results in 23 patients. Ann Thorac Surg. (2001) 72:1902–5. 10.1016/S0003-4975(01)03210-6 11789768

[22] Suda T Ashikari S Tochii D Tochii S Takagi Y. Dual-port thymectomy using subxiphoid approach. Gen Thorac Cardiovasc Surg. (2014) 62:570–2. 10.1007/s11748-013-0337-y 24170660

[23] Suda T Hachimaru A Tochii D Maeda R Tochii S Takagi Y. Video-assisted thoracoscopic thymectomy versus subxiphoid single-port thymectomy: initial results. Eur J Cardiothorac Surg. (2016) 49(Suppl. 1):i54–8. 10.1093/ejcts/ezv338 26468270

[24] Suda T Tochii D Tochii S Takagi Y. Trans-subxiphoid robotic thymectomy. Interact Cardiovasc Thorac Surg. (2015) 20:669–71. 10.1093/icvts/ivv001 25697983

[25] Ohta M Hirabayasi H Okumura M Minami M Matsuda H. Thoracoscopic thymectomy using anterior chest wall lifting method. Ann Thorac Surg. (2003) 76:1310–1. 10.1016/S0003-4975(03)00445-4 14530043

[26] Shiono H Kadota Y Hayashi A Okumura M. Comparison of outcomes after extended thymectomy for myasthenia gravis: bilateral thoracoscopic approach versus sternotomy. Surg Laparosc Endosc Percutan Tech. (2009) 19:424–7. 10.1097/SLE.0b013e3181c48242 20027081

[27] Shigemura N Shiono H Inoue M Minami M Ohta M Okumura M Inclusion of the transcervical approach in video-assisted thoracoscopic extended thymectomy (VATET) for myasthenia gravis: a prospective trial. Surg Endosc. (2006) 20:1614–8. 10.1007/s00464-005-0614-7 16794781

[28] Zahid I Sharif S Routledge T Scarci M. Video-assisted thoracoscopic surgery or transsternal thymectomy in the treatment of myasthenia gravis. Interact Cardiovasc Thorac Surg. (2011) 12(1):40–6. 10.1510/icvts.2010.251041 20943831

[29] Jurado J Javidfar J Newmark A Lavelle M Bacchetta M Gorenstein L Minimally invasive thymectomy and open thymectomy: outcome analysis of 263 patients. Ann Thorac Surg. (2012) 94:974–81.; discussion 981–2. 10.1016/j.athoracsur.2012.04.097 22748641

[30] Chen H Xu G Zheng W Chen C. Video-assisted thoracoscopic extended thymectomy using the subxiphoid approach. J Vis Surg. (2016) 2:157. 10.21037/jovs.2016.09.02 29078543 PMC5638370

[31] Hsu CP Chuang CY Hsu NY Chen CY. Comparison between the right side and subxiphoid bilateral approaches in performing video-assisted thoracoscopic extended thymectomy for myasthenia gravis. Surg Endosc. (2004) 18:821–4. 10.1007/s00464-003-9146-1 15216866

[32] O'Sullivan KE Kreaden US Hebert AE Eaton D Redmond KC. A systematic review of robotic versus open and video assisted thoracoscopic surgery (VATS) approaches for thymectomy. Ann Cardiothorac Surg. (2019) 8:174–93. 10.21037/acs.2019.02.04 31032201 PMC6462547

[33] Tu YR Lai FC Li X Lin M Duan HB Fu CG Trans-areola single port endoscopic thoracic sympathectomy for the treatment of palmar hyperhidrosis: a new surgical approach. Zhonghua Yi Xue Za Zhi. (2011) 91:3131–3. 22340656

[34] Xu K Bian W Xie H Ma H Ni B. Single-port video-assisted thoracoscopic wedge resection: novel approaches in different genders. Interact Cardiovasc Thorac Surg. (2016) 23:202–7. 10.1093/icvts/ivw119 27127103

[35] Sihoe AD Au SS Cheung ML Chow IK Chu KM Law CY Incidence of chest wall paresthesia after video-assisted thoracic surgery for primary spontaneous pneumothorax. Eur J Cardiothorac Surg. (2004) 25:1054–8. 10.1016/j.ejcts.2004.02.018 15145009

[36] Caso R Kelly CH Marshall MB. Single lumen endotracheal intubation with carbon dioxide insufflation for lung isolation in thoracic surgery. Surg Endosc. (2019) 33:3287–90. 10.1007/s00464-018-06614-9 30511311

[37] Göttgens KW Siebenga J Belgers EH van Huijstee PJ Bollen EC. Early removal of the chest tube after complete video-assisted thoracoscopic lobectomies. Eur J Cardiothorac Surg. (2011) 39:575–8. 10.1016/j.ejcts.2010.08.002 20833554

[38] Haroutiunian S Lecht S Zur AA Hoffman A Davidson E. The challenge of pain management in patients with myasthenia gravis. J Pain Palliat Care Pharmacother. (2009) 23:242–60. 10.1080/15360280903098523 19670021

